# Herbivore-Induced DNA Demethylation Changes Floral Signalling and Attractiveness to Pollinators in *Brassica rapa*

**DOI:** 10.1371/journal.pone.0166646

**Published:** 2016-11-21

**Authors:** Roman T. Kellenberger, Philipp M. Schlüter, Florian P. Schiestl

**Affiliations:** Department of Systematic and Evolutionary Botany, University of Zurich, Zurich, Switzerland; Natural Resources Canada, CANADA

## Abstract

Plants have to fine-tune their signals to optimise the trade-off between herbivore deterrence and pollinator attraction. An important mechanism in mediating plant-insect interactions is the regulation of gene expression via DNA methylation. However, the effect of herbivore-induced DNA methylation changes on pollinator-relevant plant signalling has not been systematically investigated. Here, we assessed the impact of foliar herbivory on DNA methylation and floral traits in the model crop plant *Brassica rapa*. Methylation-sensitive amplified fragment length polymorphism (MSAP) analysis showed that leaf damage by the caterpillar *Pieris brassicae* was associated with genome-wide methylation changes in both leaves and flowers of *B*. *rapa* as well as a downturn in flower number, morphology and scent. A comparison to plants with jasmonic acid-induced defence showed similar demethylation patterns in leaves, but both the floral methylome and phenotype differed significantly from *P*. *brassicae* infested plants. Standardised genome-wide demethylation with 5-azacytidine in five different *B*. *rapa* full-sib groups further resulted in a genotype-specific downturn of floral morphology and scent, which significantly reduced the attractiveness of the plants to the pollinator bee *Bombus terrestris*. These results suggest that DNA methylation plays an important role in adjusting plant signalling in response to changing insect communities.

## Introduction

In order to maximise their fitness, organisms have to invest both in their survival and in reproduction. In the case of insect-pollinated plants, there is a strong trade-off in resource investment as the two processes are often competing—not only on a metabolic, but also on an ecological level [1–3]: plant defence measures can deter pollinators [4, 5], and signals attractive to pollinators can also attract herbivores [6, 7], a phenomenon known as the defence-apparency dilemma [8]. Since herbivore and pollinator compositions can fluctuate in time and space, plants need a system to quickly react to alterations of the surrounding insect community [9]. A candidate mechanism mediating this response is the (de-) methylation of DNA cytidine residues, which can influence gene transcription in a sequence context-dependent manner [10, 11]. In recent years, DNA methylation has gained a lot of attention, since changes in the methylome have been shown to be partially heritable in plants [12, 13]. However, our understanding of the influence and relevance of DNA methylation on traits shaping defence-reproduction trade-offs is still fragmentary.

The diploid model crop plant *Brassica rapa* L. is an ideal system for studying plant-insect relationships as it is visited by a broad range of pollinator [14] and herbivore species [15]. Mediated mainly by jasmonate plant hormones, herbivory induces both direct (e.g. via leaf glucosinolates) and indirect defence reactions in Brassicaceae [16, 17]. Since glucosinolate-mediated defence has been overcome by several specialist herbivore species including the butterfly *Pieris brassicae* L. [18], signals mediating indirect defence mechanisms play an important role for herbivore deterrence in *B*. *rapa*. In this species, tissue damaged by several caterpillar species can induce volatile organic compounds (VOC), which attract plant mutualists such as parasitoid wasps [19, 20]. Herbivory also has an indirect effect on a variety of other traits, including floral signals. The onset of flowering [21], flower number [22], flower morphology, and the emission of floral VOCs [23] can be significantly altered in *B*. *rapa* plants attacked by herbivores. In some cases, these plastic changes lead to shorter or fewer visits by pollinators [23, 24], which supports the idea of a trade-off between defence and reproduction.

Swift responses of plants to environmental triggers require a fast change in gene expression. A major mechanism involved in this reaction is the reversible methylation of DNA cytidine residues, which is enzymatically catalysed by methyltransferases and often leads to an altered expression of target genes [25, 26]. Stress induced by herbivores can lead to DNA methylation changes in defence-related genes in plants [27]. Since partial retention of DNA methylation patterns during meiosis and early embryogenesis allows some of these changes to be passed on to progeny [13], DNA methylation may play a role in priming direct descendants to environmental changes experienced by the parental plants [28–30]. Several cases have been described where at least a part of herbivory-induced DNA-methylation changes were transmitted to offspring along with the observed phenotypic changes [31, 32], and one study even recorded an increased resistance to herbivory in the unexposed progeny of stressed plants [33]. Since DNA methylation changes can also dramatically alter floral phenotypes [34, 35], methylome alterations induced by herbivory could potentially influence interactions of plants with other insects such as pollinators. However, the role of herbivory-induced DNA methylation changes on pollinator-relevant phenotypic traits has not been thoroughly investigated so far.

In this study, we used methylation-sensitive amplified fragment length polymorphism (MSAP) to screen for methylome changes in *Brassica rapa* plants subjected to the specialist herbivore *Pieris brassicae* or the plant hormone methyl jasmonate (MeJA). We quantified floral phenotypic changes, compared them with a set of *B*. *rapa* genotypes demethylated with the DNA methyltransferase inhibitor 5-azacytidine [36], and assessed the impact of these changes on the pollinator *Bombus terrestris* L. [14]. Specifically, we hypothesise that a) induction of biotic and chemical defence both lead to tissue-specific DNA methylation changes accompanied by alterations of floral traits in *B*. *rapa*, b) the observed phenotypic effects are similar to chemically demethylated *B*. *rapa* plants, and c) phenotypic changes induced by methylome alterations in *B*. *rapa* are sufficient to change the pollinator attraction.

## Material and Methods

### Plants and treatments

To minimise the presence of genetic variation that could confound the analysis of DNA methylation states, we used the inbred *B*. *rapa* ssp. *trilocularis* line R-o-18 in the MSAP experiment [37]. Plants were grown from seeds in 7×7 cm pots with standard soil (Einheitserde Werkverband e.V., Germany) in climate chambers (18 h light, 21°C, 65% relative humidity) with daily watering and no fertilisation. Three days before anthesis, a total of thirty-six plants were randomly assigned to a control-, herbivory-, and MeJA group. Two fifth instar *P*. *brassicae* larvae were placed each on a mature leaf of plants from the herbivory group and allowed to feed for 24 h. The two infested leaves per plant were encaged in transparent perforated plastic bags to keep the larvae off plant reproductive parts. The treatment of the MeJA group is based on Bruinsma et al. [38]: The plant defence hormone methyl jasmonate (Sigma Aldrich, Switzerland) was diluted to a 1 mM emulsion in 0.1% Triton X-100 (Sigma Aldrich, Switzerland) and sprayed on vegetative plant parts on two consecutive days (two applications in total). Control plants were left untreated.

The DNA demethylation experiment was conducted with full-sib families generated by manual crossing of rapid-cycling *B*. *rapa* plants (Wisconsin fast plants, Wisconsin Alumni Research Foundation, WI, USA). Seeds from five of these crossings (genotypes A-E) were treated with 5-azacytidine (5-azaC, Sigma Aldrich, Switzerland) according to King (1998): Seeds were sown on filter paper in petri dishes and soaked in 5-azaC solution (0.05 mM 5-azaC, 0.5 mM 2-(*N*-morpholino)ethanosulphonic acid, pH 6.3). Since 5-azaC treatment delays the flowering time in *B*. *rapa* [39], the control group was sown two days later on filter paper with 2 ml ddH_2_O to ensure simultaneous flowering. Petri dishes were sealed and incubated in the dark at 16°C for 3 days. Seedlings were washed three times with water and transferred to soil. The plants were grown under the same conditions as the plants for the MSAP experiment. All stunted and damaged plants were removed, and the final sample size was balanced to 200 plants (20 plants × 5 genotypes × 2 treatments) by random removal of excess plants.

### DNA extraction and MSAP generation

Treated leaves and (untreated) flowers from the R-o-18 plants were collected two weeks after treatment, flash frozen in liquid nitrogen and stored at -80°C. DNA was extracted using a Qiagen DNeasy Plant Mini Kit (Qiagen, CA, USA) and the manufacturer’s protocol, quantified with a Qubit 2.0 fluorometer using a dsDNA-HS assay kit (Thermo Fisher Scientific Inc., CA, USA) and visually checked on a 1.2% agarose gel. Generation of MSAP fragments was performed after Xiong et al. (1999) [40] with some modifications. The full protocol is provided in the Supporting Information; enzymes were obtained from New England Biolabs, MA, USA and from Thermo Fisher Scientific Inc., CA, USA. This protocol uses the enzyme combinations *Eco*RI–*Hpa*II and *Eco*RI–*Msp*I respectively. While *Hpa*II and *Msp*I are isoschizomers recognising the sequence 5’-CCGG-3’, *Hpa*II is sensitive to double-stranded methylation of the internal cytosine, and *Msp*I to single-, or double-stranded methylation of the external cytosine [41]. Fragments were selectively amplified using four FAM or HEX-labelled primer pairs. One μl selective amplification of each sample was mixed with 10 μl size standard (LIZ 600, Applied Biosystems Inc., CA, USA), diluted 1:100 in Hi-Di-formamide (Applied Biosystems Inc., CA, USA), and denatured for 3.5 min at 92°C. Fragments were separated on an ABI 3130*xl* sequencer (Applied Biosystems Inc., CA, USA) using the manufacturer's protocols.

### MSAP scoring and analysis

The generated MSAP profiles were analysed with GeneMapper v. 4.1 (2009, Thermo Fisher Scientific Inc., CA, USA). Fragments between 50 and 500 bp were included for scoring. Several precautions were taken to ensure reproducibility of the results [42]: a) Negative control samples (without DNA) were included in all PCR steps. b) All samples were fully randomised and blindly scored by the same person. c) Loci with electropherogram peaks of less than 100 relative fluorescent units, merged and unclean peaks, peaks occurring in less than 2 samples, and peaks occurring in the negative control samples were removed from the dataset. d) The whole MSAP generation and analysis was repeated from DNA extraction for 17% of all samples (two samples × treatment × tissue). The total scoring error rate was calculated as the ratio of markers scored differently in the replicate samples relative to the total amount of scored markers in the dataset. The MSAP data was analysed with the R package *msap* v. 1.1.4 [43]: a locus with both *Eco*RI-*Hpa*II, and *Eco*RI-*Msp*I bands present (1/1) was considered unmethylated, a locus with an absent *Eco*RI-*Msp*I band (1/0) externally methylated (single-strand methylation of the external cytosine), and a locus with an absent *Eco*RI-*Hpa*II band (0/1) internally methylated (double or single strand methylation of the internal cytosine). Since the *B*. *rapa* line (R-o-18) used in this study is highly inbred, loci with absence of both bands (0/0, but present in other individuals in the dataset) were scored as hypermethylated (methylated internal and external cytosines of both strands; see [44] for another example). The *msap* package estimates the amount of epigenetic variation based on the Shannon diversity index calculated within each locus. Epigenetic differentiation was computed with principal component analysis (PCA) using the R packages *caret* v. 6.0–68 and *ggbiplot* v. 0.55, and differences between groups were calculated with pair-wise analysis of molecular variance (AMOVA) with 10000 permutations [43]. In addition, locus-by-locus AMOVA with 10000 permutations was conducted with the R package *mmod* v. 1.3.1 to determine the number of loci with significant methylation differentiation, and the ratio between newly demethylated and methylated loci was assessed with a two-sided χ^2^-test [45].

### Morphological and floral volatile analysis

Both phenotypic traits and floral VOCs were collected for all plants individually three days after anthesis. Plant height, flower diameter, and number of leaves, buds, and flowers were recorded. Spacing between flowers and pedicel length of three flowers per plant were measured to assess inflorescence density. For the 5-azaC-treated plants, additional traits including petal surface (4 × π × petal width × petal length), nectar volume and pollen quantity were measured. Anthers from three flowers per plant were collected in 600 μl ddH_2_O containing 0.4% Tween 80 (Sigma Aldrich, Switzerland) and pollen was counted on a Cell Lab Quanta flow cytometer with a mercury arc lamp (Beckman Coulter, CA, USA) [46].

Flower VOC were collected with non-destructive headspace sorption from 10:00 to 12:00 before the phenotypic measurements. Whole inflorescences were enclosed in glass cylinders treated with Sigmacote (Sigma Aldrich, Switzerland) and sealed with Teflon plates around the peduncle. Clean air was pushed through active charcoal filters into the cylinders at a flow rate of 120 ml min^-1^ for 2 h. Simultaneously, air was pulled out of the cylinders through glass tubes loaded with 20 mg Tenax TA (60/80 mesh, Supelco, Bellefonte, PA, USA) at the same flow rate and duration. Air samples from empty glass cylinders were used as controls. VOC samples were analysed using gas chromatography with mass selective detection (GC-MSD) as described in ref. [23]. Compounds were identified and quantified with a calibrated mass spectral library built on authentic reference standards [23]. Non-identifiable VOC as well as VOC with an amount below the mean air-control level in > 10% of samples were excluded from the dataset. VOC quantities were calculated in pg flower^-1^ l^-1^ sampled air. All analyses were done in the Agilent MSD ChemStation program E. 02.02 (2011).

### Bioassays

The attraction of pollinators to 5-azacytidine treated plants was determined in dual-choice bioassays with bumblebees (*Bombus terrestris*, Biobest Group, Belgium). All bioassays were conducted one day after VOC collection to avoid any bias from plant handling. Before the assay, the bees were allowed to forage on untreated *B*. *rapa* plants from all genotypes for 2 h. Subsequently, one pair consisting of a randomly chosen control, and treated plant of the same genotype was placed with 20 cm distance in a flight cage (2.5 m length, 1.8 m width, 1.2 m height). Bees were released individually in the cage. After the first landing on a flower, the chosen plant was recorded and the bee was removed from the experiment. After a sequence of six visits, all bees were returned to their hive box, the plant pair was removed and a new pair was installed switching the position of control-, and treated plant. A total of 22 plant pairs and one *B*. *terrestris* nest box were used in this experiment.

### Statistics

Treatment effects were analysed independently for the MSAP and 5-azacytidine experiments. Prior to both analyses, response variables were Box-Cox transformed [47], normality was examined with a Shapiro-Wilk test [48], and homoscedasticity was assessed using Fligner-Killeen’s test [49]. Treatment effects were assessed first on variation of trait classes using multivariate analysis of variance (MANOVA). Morphological variables were combined in one class, and scent compounds were grouped according to chemical classes (aromatics, terpenoids, fatty acid derivatives, nitrogen compounds, and all VOC together), as they partially share biosynthetic pathways [50]. For the MSAP experiment, treatment effects within significant MANOVA classes were calculated using a one-way ANOVA, and multiple comparisons between treatments were performed with *post-hoc* pair-wise *t*-tests. For the 5-azacytidine experiment, treatment, genotype and treatment × genotype effects on single variables within significant MANOVA groups were calculated using a two-way ANOVA, and multiple comparisons among plant families were performed with *post-hoc* pair-wise *t*-tests with Bonferroni correction. The effect of the 5-azaC treatment on pollinator attraction was calculated globally using a binomial test, and treatment × genotype effects were assessed with repeated measures ANOVA. All statistical analyses were carried out in *R* v. 3.0.2 (R Development Core Team 2013) with the package *MASS* v. 7.3–35 (Venables and Ripley 2002).

## Results

### Alterations of MSAP profiles upon herbivory and methyl jasmonate treatment

**Fig 1:**
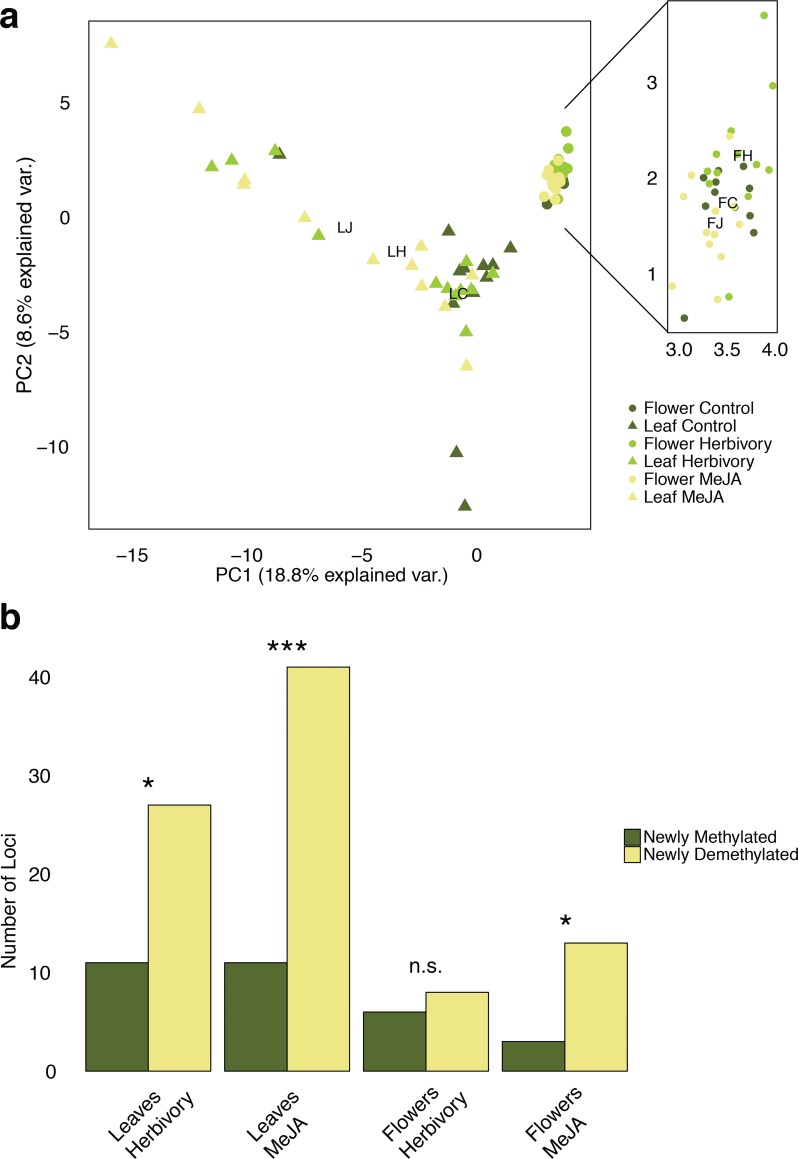
Treatment effects on DNA methylation Fig 1a: PCA of differentiation in methylation sensitive loci state shows a clear separation of samples from leaves and flowers. Methylome changes upon the different treatments (control, herbivory, and MeJA) were significant in both leaves and flowers, but much more prominent in leaf tissue than in flower tissue. Centroid positions of the control -, herbivory-, and MeJA group are indicated with LC, LH, and LJ for the leaf samples, and with FC, FH, and FJ respectively for the flower samples. Fig 1b: Number of loci detected with locus-by-locus AMOVA in both tissues and treatments with either a significant methylation gain (change from an unmethylated to an external, internal, or hypermethylated state as well as change from an external or internal to a hypermethylated state) or methylation loss (change from an external or internal to an unmethylated state as well as change from a hypermethylated to an external, internal, or unmethylated state). Results of the two-sided χ^2^-tests: Leaves Herbivory: χ^2^ = 5.921, P = 0.015; Leaves MeJA: χ^2^ = 16.173, P<0.001; Flowers Herbivory: χ^2^ = 0.071, P = 0.789; Flowers MeJA: χ^2^ = 5.063, P = 0.024 (α = 0.05).

Using MSAP, we screened for DNA methylation changes in leaf and flower tissue of herbivore and MeJA-treated *Brassica rapa* R-o-18 plants. The four selective primer combinations amplified a total of 297 fragments between 50 and 500 bp. Of these markers, 295 were susceptible to methylation (proportion of a particular observed *Hpa*II/*Msp*I pattern >5%), and 85 of them were polymorphic in the sampled individuals (29%). A marker was considered polymorphic if both methylated and unmethylated states occurred at least twice across all samples. The remaining two markers were unmethylated, both being polymorphic (S1 Table). With 4.91%, the observed scoring error rate lay within the reported range of ≤ 5% [42]. The mean Shannon diversity index for methylation-susceptible loci (0.37) was not significantly higher than for unmethylated loci (0.23, Wilcoxon rank sum test: *W* = 117.5, *P* = 0.363). Results from AMOVA and PCA indicate an inherent methylome difference between leaf and flower tissue within a single individual (Table 1, Fig 1A). In addition, significant induced methylome differentiation was found between all investigated treatments, except between leaves of herbivore and MeJA-treated plants (Table 1). In leaves, both herbivory and MeJA application resulted in a significantly higher proportion of demethylation events across all differentially methylated loci (Fig 1B). In general, treatment effects in flowers were weaker (Fig 1A), and a significant demethylation was only observed for the MeJA treatment (Fig 1B).

**Table 1:** Epigenetic differentiation between leaf and flower tissue of Brassica rapa R-o-18 plants after herbivory and MeJA treatment.

Pair-wise AMOVA of meth. markers	Control Flower	Control Leaf	Herbivory Flower	Herbivory Leaf	MeJA Flower
Control Leaf	F = 0.21 **P<0.0001**				
Herbivory Flower	F = 0.02 **P = 0.0007**	F = 0.24 **P<0.0001**			
Herbivory Leaf	F = 0.31 **P<0.0001**	F = 0.06 **P = 0.0244**	F = 0.34 **P<0.0001**		
MeJA Flower	F = 0.02 **P = 0.0008**	F = 0.19 **P<0.0001**	F = 0.03 **P = 0.0006**	F = 0.31 **P<0.0001**	
MeJA Leaf	F = 0.38 **P<0.0001**	F = 0.11 **P = 0.0044**	F = 0.38 **P<0.0001**	F = 0.03 P = 0.1153	F = 0.37 **P<0.0001**
**Global AMOVA of meth. markers**	**Deg. of freedom among groups**	**Deg. of freedom within groups**	**Variance among groups**	**Variance within groups**	**F-value**
	5	66	0.02	0.08	F = 0.23 **P<0.0001**

F- and P-values for global and pair-wise AMOVA of methylation-susceptible loci from different tissues (leaves and flowers) of control-, herbivory-, and MeJA-treated plants show a larger epigenetic differentiation between the tissues, and a smaller but still significant epigenetic differentiation between the different treatments except for leaves of herbivory- and MeJA-treated plants. *P* value of significant differentiations in bold (α = 0.05).

### Phenotypic effects of herbivory and methyl jasmonate treatments

**Fig 2:**
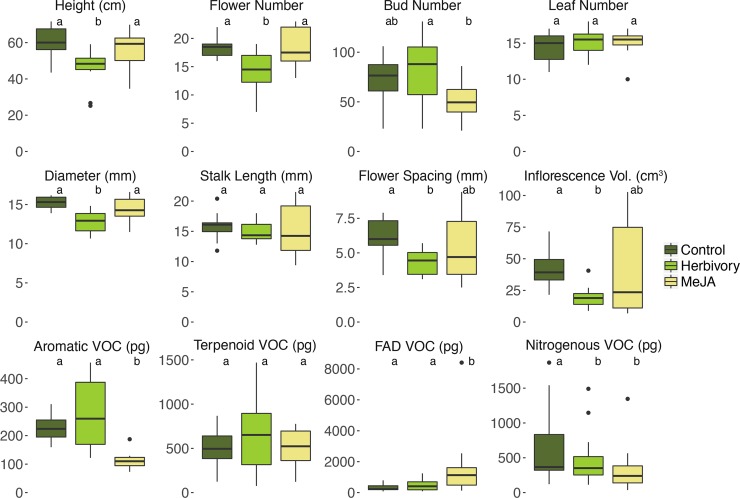
Treatment effects on morphology and floral volatiles Boxplots showing the effect of the herbivory and MeJA treatment on morphological traits and the emission of the main VOC classes in *B*. *rapa*. Herbivory led to a decrease in morphological traits (plant height, flower number, diameter, and spacing, and inflorescence volume) and nitrogenous VOC, while MeJA application led to a significant change in the cumulative emission of total aromatic, fatty acid derivatives (FAD), and nitrogenous VOC. Letters (a, b) above boxes indicate different significance groups (α = 0.05).

We compared both floral morphology and floral volatiles (VOC) between the three different *B*. *rapa* treatment groups. Treatment effects could be measured on overall floral morphology, the entire VOC production, and all different compound classes (S2 Table, Fig 2). Five phenotypic traits as well as eight VOC of all compound classes were significantly reduced (S2 Table). However, herbivory and MeJA had a different impact on morphology and VOC (Table 2, Fig 2): While herbivory caused a decrease of six morphological traits, all but two nitrogenous VOC were unaffected. In MeJA-treated plants, no morphological changes were detected, but the emission of eight VOC distributed over all compound classes was significantly lower. *Z*-3-hexenyl acetate was the only VOC with an increased emission in MeJA-treated plants.

**Table 2:** Phenotypic changes of *B*. *rapa* R-o-18 plants after herbivory and MeJA treatment.

	Control—Herbivory		Control—MeJA		Herbivory—MeJA	
Plant trait	change	*P* value	Change	*P* value	change	*P* value
Plant Height	↓	**0.002**	-	0.239	↑	**0.027**
Bud Number	-	0.417	-	0.176	↓	**0.044**
Flower Number	↓	**0.006**	-	0.874	↑	**0.006**
Flower Diameter	↓	**< 0.001**	-	0.085	↑	**0.011**
Flower Spacing	↓	**0.041**	-	0.290	-	0.290
Inflorescence Volume	↓	**0.016**	-	0.209	-	0.209
*p*-Anisaldehyde	-	0.420	↓	**< 0.001**	↓	**< 0.001**
Benzaldehyde	-	0.380	↓	**< 0.001**	↓	**< 0.001**
Methylbenzoate	-	0.970	↓	**< 0.001**	↓	**< 0.001**
Camphor	-	0.753	↓	**< 0.001**	↓	**0.001**
*Z*-α-Farnesene	-	0.292	↓	**0.014**	-	0.117
*Z*-3-Hexenyl acetate	-	0.158	↑	**< 0.001**	↑	**0.003**
Benzylnitrile	↓	**0.013**	↓	**< 0.001**	↓	**0.001**
Methylanthranilate	↓	**< 0.001**	↓	**< 0.001**	↓	**0.001**

*Post-hoc* multiple comparisons show a different impact of herbivory and MeJA on the *B*. *rapa* R-o-18 phenotype. While herbivory treatment mainly decreased morphological traits, the application of MeJA changed floral volatile emission. Emission of the nitrogenous compounds benzylnitrile and methylanthranilate was reduced under both treatments. Arrow up: trait increase, arrow down: trait decrease, dash: no trait change, *P* value of significant changes in bold (α = 0.05).

### Genotype-specific effects of DNA demethylation on morphology and VOC

**Fig 3:**
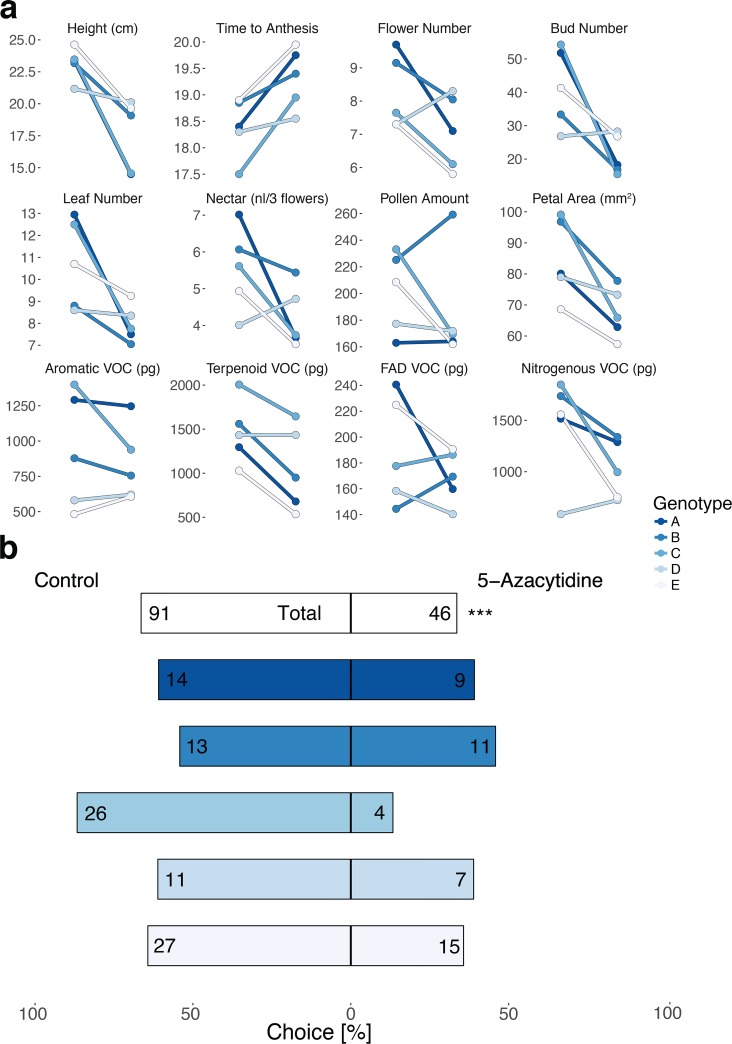
Effect of DNA demethylation on plant morphology, floral volatiles, and pollinator choice Fig 3a: Plots showing treatment × genotype interactions on morphological traits and floral volatiles (VOC). A comparison of trait divergence between treatment (left points in each line plot) and control group (right points) across all plant genotypes shows pronounced treatment × genotype interactions for all traits (lines connecting both points; e.g. stronger treatment effects in genotype C, and weaker effects in genotype D). Significant treatment effects are indicated in S3 and S4 Tables. Fig 3b: Barplot showing the choice of *B*. *terrestris* in the two-choice assays between control and 5-azaC treatment (percentage and total choices) for all plants as well as for individual genotypes. In total, control plants were favoured over 5-azaC-treated plants with 66.4% to 33.6% landings (top bar with significance asterisks, α = 0.05). The preference for control plants was dependent on plant genotype and very pronounced in genotype C (bars below).

We assessed the effect of 5-azacytidine induced DNA demethylation on morphological traits and VOC production of five *B*. *rapa* full-sib families. 5-azacytidine is a cytidine analogue, which is incorporated into DNA during replication and inhibits DNA methyltransferases [51]. Genome demethylation with 5-azaC had a significant, genotype-specific impact on overall plant morphology (S3 Table) as well as the entire floral scent bouquet (S3 Table, Fig 3A). While 5-azaC-treatment decreased all morphological traits (except pollen quantity) across all plant genotypes, the impact of the treatment was highly variable among the different VOC (S4 Table, Fig 3A). Genotype × treatment interactions were present in all morphological traits, but only in aromatic VOC (S4 Table, Fig 3A). Specifically, the results of the pair-wise *t*-tests (Table 3) show considerable morphological variation among genotypes in susceptibility to the 5-azaC-treatment. Genotype as a single factor was significant for all morphological traits except pollen quantity as well as all floral scent compounds (S3 Table), confirming the presence of inherent genotype-specific differences in plant morphology and VOC production.

**Table 3:** Phenotypic changes in different genotypes of rapid-cycling *B*. *rapa* plants after 5-azaC treatment. (*post-hoc* multiple phenotypic comparisons).

	Genotype A		Genotype B		Genotype C		Genotype D		Genotype E	
Plant trait	change	*P* value	change	*P* value	change	*P* value	change	*P* value	change	*P* value
Plant Height [cm]	↓	**< 0.001**	↓	**< 0.001**	↓	**< 0.001**	-	0.310	↓	**0.001**
Time to Flowering [d]	↑	**< 0.001**	-	0.109	↑	**< 0.001**	-	0.373	-	**0.017**
Leaf Number	↓	**< 0.001**	↓	**0.022**	↓	**< 0.001**	-	0.597	-	0.158
Bud Number	↓	**< 0.001**	↓	**< 0.001**	↓	**< 0.001**	-	0.705	↓	**0.004**
Flower Number	↓	**0.013**	-	0.140	-	0.107	-	0.140	↓	**0.022**
Flower Petal Area [mm^2^]	↓	**< 0.001**	↓	**< 0.001**	↓	**< 0.001**	↓	**0.022**	↓	**0.022**
Plant Petal Area [mm^2^]	↓	**< 0.001**	↓	**< 0.001**	↓	**< 0.001**	-	0.338	↓	**0.002**
Nectar Amount [μl]	↓	**< 0.001**	-	0.948	↓	**0.019**	-	0.948	-	0.058
Phenylethyl Alcohol	-	1.000	-	0.196	↓	**0.001**	-	1.000	-	1.000

Multiple comparisons show a decreased phenotypic performance in response to genome demethylation in all five different plant families (genotype A-E). However, the five genotypes differ in their susceptibility to the 5-azaC-treatment. While some genotypes (e.g. genotype C) exhibit phenotypic changes in a large number of traits, other genotypes such as genotype D are almost unaffected. Arrow up: trait increase, arrow down: trait decrease, dash: no trait change, *P* value of significant changes in bold (α = 0.05).

### Attractiveness of demethylated plants to bumblebees

We used dual-choice assays to determine whether the cumulative effect of the observed phenotypic changes in demethylated plants is strong enough to affect the attraction of pollinators. Overall, 5-azaC-treated plants were significantly less attractive to bumblebees (33.6% of all landings, Fig 3B). A comparison between plant genotypes showed a different magnitude of deviation from a 1:1 ratio (genotype A: 39.1% landings, B: 45.8%, C: 13.3%, D: 38.9%, E: 35.7%). However, statistical analysis did not reveal any interaction of treatment and genotype (Fig 3B).

## Discussion

Using the model crop plant species *B*. *rapa*, we investigated the role of DNA methylation in floral signalling in response to herbivory. Our results showed that foliar herbivory of *P*. *brassicae* caterpillars leads to genome-wide methylation changes not only in the leaves, but also in the undamaged flowers of *B*. *rapa*. A chemical induction of plant defence resulted in similar demethylation patterns in leaves, but significant differences both in the methylome and phenotype of flowers. The observed methylome changes are thus likely stress-specific and may have the potential to be transmitted to the next generation. Treatment of *B*. *rapa* with 5-azaC further showed that floral changes observed upon DNA demethylation are correlated with a significant decrease in the attractiveness of the plants to their main pollinator *B*. *terrestris* [14].

### DNA demethylation upon induction of plant defence

In plants, an average of around 80% of CpG sites are methylated in a tissue-specific pattern [52, 53], playing an important role in the regulation of gene activity and immobilisation of transposable elements [54]. The MSAP profiles obtained from the *B*. *rapa* plants in this study are in agreement with these observations as they show a high proportion of methylated CpG-sites with considerable variation between leaf and flower tissue. Biotic and abiotic stresses have been shown to induce changes in plant methylomes [27, 31], and differences in biotic damage could also be linked to variation in DNA methylation [55]. In this study, the stress treatments led to a net shift towards partial or even a complete loss of cytosine methylation. Genome-wide demethylation has been observed in a range of plant systems under various stresses such as high salinity, low temperatures [56], or viral infection [57], and is usually associated with the up-regulation of stress-response genes [58]. However, the overall picture is far less clear-cut and several other studies have also observed DNA hypermethylation [59, 60] or no clear trend at all [61]. In the genus *Brassica*, it has been shown that leaf herbivory by *Pieris* activates the jasmonate signal pathway [38], and spraying *Brassica* plants with MeJA induces defence reactions in leaves [62]. This may explain our finding that the methylome in leaf tissue was altered upon treatment, but not significantly different between herbivory and MeJA-treated plants. On the other hand, DNA methylation patterns in flower tissue were different between all three groups, suggesting a stress-specific response to both treatments.

### Alterations of floral signals upon herbivory

Fine-tuning of signals such as floral shape, colour, and scent is crucial for the fitness of insect-pollinated plants [63]. While it has been shown that herbivory can indeed lead to pollinator-relevant changes in floral signalling [64–66], results from studies with different plant- and herbivore systems are often very heterogeneous. In *B*. *rapa*, previous work has not only documented an altered morphology and VOC emission in both leaves and flowers under herbivore attack, but also identified a trade-off between indirect defence and pollinator attraction [23]. Here, we found that herbivory by *P*. *brassicae* resulted in a net decrease among the measured values of eight traits, many of which are highly relevant to determining plant attractiveness to insects [67–69]. However, some of these effects contrast with the findings of Schiestl et al. [23], where herbivory induced a more pronounced decrease in floral volatiles and an increase in the number of open flowers, which is probably due to different *B*. *rapa* subspecies used in the two studies (*B*. *rapa* ssp. *oleifera* in Schiestl et al., and *B*. *rapa* ssp. *trilocularis* in this study). As reflected by methylome differentiation, effects on floral traits differed significantly between *P*. *brassicae* and MeJA-treated plants: While the emission of several floral volatiles was more strongly reduced under MeJA treatment, herbivory had a much greater impact on multiple morphological traits. Although it has been shown that plastic responses of *B*. *rapa* can vary specifically between different types of herbivory [70], explaining these differences is not straightforward, since a) MeJA may have additional effects beyond plant resistance [71], b) although the application of 1mM MeJA has been shown to attract parasitoids in *B*. *oleracea* [38], we cannot exclude that this dosage may be on the upper limit of the physiological range, and c) plant reactions to continuous herbivore feeding may be different from reactions to a two-times application of MeJA. A complete understanding of the underlying causes therefore requires additional experiments with varying intensity of both treatments.

### Association of DNA demethylation, signalling changes and pollinator choice

Results from this study show that foliar herbivory is associated with both DNA demethylation and floral phenotypic changes. However, it remains challenging to establish a causative link between methylome changes and phenotypic responses since a) methylome structure is not completely independent from genetic variation [55], and b) other processes such as RNA interference may also induce phenotypic responses in plants [72]. Several studies have disentangled effects induced by methylome changes from other causes by either comparing methylomes of different organs within single plant individuals [34, 73], or introduction of DNA methylation changes with chemicals such as 5-azaC [36, 74, 75]. There are some caveats in interpreting the results of 5-azaC experiments: Its action is stochastic and unspecific, and although the application is restricted to a short time during germination, it also incorporates into RNA [76], which may cause additional effects such as the observed shift in flowering time. However, its impact on DNA methylation is well-documented at the molecular level, and it is useful for assessing the phenotypic impact of DNA demethylation across different genetic backgrounds [77]. Our finding of demethylation with 5-azaC resulting in a reduced phenotypic expression has been documented in several other studies, including an earlier screen of 5-azaC treated *B*. *rapa* R-o-18 plants [39]. As in the *P*. *brassicae-*infested plants, the impact of genomic demethylation was more severe on floral morphology than on volatile production. This could reflect that the complex regulation of polygenic morphological traits [78] may be more exposed to stochastic methylome changes than the regulation of secondary metabolites such as floral scent compounds (see [79] for a review). While the effect of demethylation on individual traits was consistent among all five genotypes, some genotypes were considerably more affected than others. Bossdorf et al. (2010) [77] showed that such differences in demethylation responses are only partially related to genetic distance, which implies that methylome variation is indeed partially independent from genetic differences. As a consequence, the attractiveness of demethylated plants to the pollinator *B*. *terrestris* tended to be weaker for more affected genotypes, although this relationship was not statistically significant. However, genomic demethylation induced by 5-azaC was sufficient to significantly reduce the overall attractiveness of the treated plants to *B*. *terrestris*. This result implies that DNA methylation changes *per se* can have a significant impact on plant signalling traits, modulating plant-insect interactions with potential fitness consequences.

In conclusion, our results indicate a strong correlation of DNA methylation states with pollinator-relevant floral traits, which can be selectively altered upon interactions with herbivores. DNA methylation thus has the potential to mediate and interconnect multiple plant-insect interactions through phenotypic plasticity, allowing a quick response to changes in the surrounding insect community. Since flowers are reproductive units, the observed DNA methylation changes may possibly be transmitted to subsequent generations [13]. Several studies have indeed shown that stress-induced DNA methylation changes can at least be partially inherited [31, 33].

## Supporting Information

S1 Protocol Protocol for methylation-sensitive amplification polymorphism assay. (DOCX)

S1 Table Summary of the MSAP analysis on *B*. *rapa* R-o-18. (DOCX)

S2 Table Phenotypic differences between *B*. *rapa* R-o-18 plants under control, herbivory, and MeJA treatment. (DOCX)

S3 Table Phenotypic changes of trait classes in rapid-cycling *B*. *rapa* plants after 5-azaC treatment. (DOCX)

S4 Table Phenotypic differences between rapid-cycling *Brassica rapa* plants under control and 5-azacytidine treatment. (DOCX)
