# Supplementary material for: Herbivore-Induced DNA Demethylation Changes Floral Signalling and Attractiveness to Pollinators in Brassica rapa
Source: PLoS One. 2016 Nov 21;11(11):e0166646. doi: 10.1371/journal.pone.0166646 (PMC5117703; doi:10.1371/journal.pone.0166646)
Supplement: S1 Protocol — (DOCX) [file pone.0166646.s001.docx]

**S1 Protocol. Methylation-sensitive amplification polymorphism assay**

| **Sequences of primer and adapters used for the MSAP analysis** | |
| --- | --- |
| **Adapter sequences** |  |
| *Eco*RI – adapter 1 | 5’ CTCGTAGACTGCGTACC |
| *Eco*RI – adapter 2 | 5’ AATTGGTACGCAGTCTAC |
| *Hpa*II/ *Msp*I – adapter 1 | 5’ GATCATGAGTCCTGCT |
| *Hpa*II/ *Msp*I – adapter 2 | 5’ CGAGCAGGACTCATGA |
|  |  |
| **Pre-amplification primer** |  |
| *Eco*RI – preamp | 5’ GACTGCGTACCAATTC |
| *Hpa*II/ *Msp*I – preamp | 5’ ATCATGAGTCCTGCTCGG |
|  |  |
| **Selective primer** |  |
| HEX - *Eco*RI – CAA | 5’ GACTGCGTACCAATTCCAA |
| FAM - *Eco*RI – ATG | 5’ GACTGCGTACCAATTCATG |
| *Hpa*II/ *Msp*I – CAA | 5’ ATCATGAGTCCTGCTCGGCAA |
| *Hpa*II/ *Msp*I – TAG | 5’ ATCATGAGTCCTGCTCGGTAG |

| **DNA restriction reactions and conditions** | | |
| --- | --- | --- |
| ***Eco*RI *– Hpa*II restriction** |  | |
| NEB buffer 1 | 10× | |
| *Eco*RI | 4 U | |
| *Hpa*II | 4 U | |
| DNA | 150 ng | |
| ddH_2_O | to 40 μl volume | |
|  |  | |
| ***Eco*RI *– Msp*I restriction** |  | |
| NEB buffer 2 | 10× | |
| *Eco*RI | 4 U | |
| *Msp*I | 4 U | |
| DNA | 150 ng | |
| ddH_2_O | to 40 μl volume | |
|  |  | |
| **Incubation** | **Time (min)** | **Temperature (°C)** |
| Restriction | 120 | 37 |

| **Adapter dimerisation reactions and conditions** | | |
| --- | --- | --- |
| ***Eco*RI Adapter dimerisation** | | |
| NEB buffer 2 | 10× | |
| *Eco*RI – adapter 1 | to 5 μM | |
| *Eco*RI – adapter 2 | to 5 μM | |
| ddH_2_O | to 30 μl volume | |
|  |  | |
| ***Hpa*II/*Msp*I Adapter dimerisation** | | |
| NEB buffer 2 | 10× | |
| *Hpa*II/*Msp*I – adapter 1 | to 50 μM | |
| *Hpa*II/*Msp*I – adapter 2 | to 50 μM | |
| ddH_2_O | to 30 μl volume | |
|  |  | |
| **Incubation** | **Time (min)** | **Temperature (°C)** |
| Denaturation | 5 | 95 |
| Annealing | 120 | 95 – 25 (touch-down) |

| **Adapter ligation reaction and conditions** | | |
| --- | --- | --- |
| **Adapter ligation** |  | |
| NEB buffer 2 | 10× | |
| *Eco*RI – adapter (Table S3) | 3 μl | |
| *Hpa*II/*Msp*I – adapter (Table S3) | 3 μl | |
| T4 DNA ligase | 20 U | |
| ATP (100 μM) | 0.5 μl | |
| BSA (10 mg/ml) | 0.1 μl | |
| ddH_2_O | to 10 μl volume | |
| Digested DNA (Table S2) | 40 μl | |
|  |  | |
| **Incubation** | **Time (min)** | **Temperature (°C)** |
| Ligation | 180 | 25 |
| Denaturation | 10 | 65 |

| **Pre-selective PCR reaction and conditions** | | |
| --- | --- | --- |
| **Pre-selective PCR** |  | |
| Dream Taq PCR MM | 1× | |
| *Eco*RI pre-selective primer | to 35 μM | |
| *Hpa*II/*Msp*I pre-selective primer | to 35 μM | |
| Ligated DNA (Table S4) | 2 μl | |
| ddH_2_O | to 25 μl volume | |
|  |  | |
| **Pre-selective PCR cycling** | **Time (min)** | **Temperature (°C)** |
| Denaturation | 3 | 94 |
| 20 cycles of: |  |  |
| Denaturation | 0.5 | 94 |
| Annealing | 0.5 | 60 |
| Elongation | 1 | 72 |
| Final extension | 1 | 72 |

| **Selective PCR reaction and conditions** | | |
| --- | --- | --- |
| **Selective PCR** |  | |
| Dream Taq PCR MM | 1× | |
| *Eco*RI FAM/HEX labeled primer | to 1.5 μM | |
| *Hpa*II/*Msp*I selective primer | to 4.5 μM | |
| DNA pre-selection (Table S5) | 5 μl (diluted 1:20 in 1× TE) | |
| ddH_2_O | to 20 μl volume | |
|  |  | |
| **Selective PCR cycling** | **Time (min)** | **Temperature (°C)** |
| Denaturation | 3 | 94 |
| 10 touch-down cycles of: |  |  |
| Denaturation | 0.5 | 94 |
| Annealing | 0.5 | 65 – 56 (1°C reduction/cycle) |
| Elongation | 1 | 72 |
| Denaturation | 0.5 | 94 |
| 25 cycles of: |  |  |
| Denaturation | 0.5 | 94 |
| Annealing | 0.5 | 54 |
| Elongation | 1 | 72 |
| Final extension | 1 | 72 |
