# Supplementary material for: Herbivore-Induced DNA Demethylation Changes Floral Signalling and Attractiveness to Pollinators in Brassica rapa
Source: PLoS One. 2016 Nov 21;11(11):e0166646. doi: 10.1371/journal.pone.0166646 (PMC5117703; doi:10.1371/journal.pone.0166646)
Supplement: S1 Table — (DOCX) [file pone.0166646.s002.docx]

**S1 Table. Summary of the MSAP analysis on *B. rapa* R-o-18.**

|  |  |  | **Methylation susceptible markers** | | **Non-methylated markers** | |
| --- | --- | --- | --- | --- | --- | --- |
| **Primer pair** | **Total markers** | **Scoring error rate** | **Total number** | **Polymorphic** | **Total number** | **Polymorphic** |
| *Eco-*ATG / *HM-*CAA | 63 | 4.37% | 62 | 17 (27%) | 1 | 1 (100%) |
| *Eco-*ATG / *HM-*TGC | 96 | 5.92% | 95 | 24 (25%) | 1 | 1 (100%) |
| *Eco-*CAA / *HM-*CAA | 58 | 3.23% | 58 | 23 (40%) | 0 | - |
| *Eco-*CAA / *HM-*TGC | 80 | 5.42% | 80 | 21 (26%) | 0 | - |
| Total | 297 | 4.91% | 295 | 85 (29%) | 2 | 2 (100%) |
| **Shannon’s diversity index ± 1 SD** | |  | 0.37 ± 0.20 | | 0.23 ± 0.03 | |

Used primer pairs, markers between 50 and 500 bp amplified from these primers, scoring error rates (calculated from repeated analysis of 17 % of samples), and frequency, polymorphisms, and calculated Shannon’s diversity indices of methylation susceptible and non-methylated markers obtained from leaves and flowers of the total 36 *B. rapa* plants (12 plants per treatment).
