# Supplementary material for: Herbivore-Induced DNA Demethylation Changes Floral Signalling and Attractiveness to Pollinators in Brassica rapa
Source: PLoS One. 2016 Nov 21;11(11):e0166646. doi: 10.1371/journal.pone.0166646 (PMC5117703; doi:10.1371/journal.pone.0166646)
Supplement: S2 Table — (DOCX) [file pone.0166646.s003.docx]

**S2 Table. Phenotypic differences between *B. rapa* R-o-18 plants under control, herbivory, and MeJA treatment.**

|  | **MANOVA** | | **ANOVA** | | **Mean ± 1 SE** | | |
| --- | --- | --- | --- | --- | --- | --- | --- |
| **Plant trait** | ***F* value** | ***P* value** | ***F* value** | ***P* value** | **Control** | **Herbivory** | **MeJA** |
| **Morphological Traits** | 3.630 | **< .001** |  |  |  |  |  |
| Plant Height [cm] |  |  | 7.602 | **0.002** | 60.76 ± 2.45 | 45.84 ± 2.90 | 56.16 ± 3.19 |
| Leaf Number |  |  | 0.974 | 0.388 | 14.33 ± 0.62 | 15.42 ± 0.51 | 15.00 ± 0.52 |
| Bud Number |  |  | 3.473 | **0.043** | 71.25 ± 6.85 | 81.67 ± 10.34 | 51.67 ± 5.79 |
| Flower Number |  |  | 7.083 | **0.003** | 18.42 ± 0.53 | 13.92 ± 1.12 | 18.08 ± 0.98 |
| Flower Diameter [mm] |  |  | 11.470 | **< 0.001** | 15.25 ± 0.21 | 12.84 ± 0.38 | 14.53 ± 0.45 |
| Flower Spacing [mm] |  |  | 3.427 | **0.044** | 6.06 ± 0.40 | 4.32 ± 0.26 | 5.26 ± 0.65 |
| Flower Stalk Length [mm] |  |  | 0.301 | 0.742 | 15.81 ± 0.64 | 14.86 ± 0.44 | 15.42 ± 1.26 |
| Inflorescence vol. [cm^3^] |  |  | 4.441 | **0.020** | 41.87 ± 4.36 | 19.42 ± 2.49 | 41.70 ± 10.07 |
| **VOC** [pg / flower l^-1^] | 19.000 | **< 0.001** |  |  |  |  |  |
| **Aromatics** | 4.458 | **< 0.001** |  |  |  |  |  |
| *p*-Anisaldehyde |  |  | 34.420 | **< 0.001** | 16.28 ± 1.40 | 19.54 ± 2.66 | 5.63 ± 0.66 |
| Benzaldehyde |  |  | 21.760 | **< 0.001** | 172.4 ± 12.8 | 219.1 ± 29.2 | 83.81 ± 8.22 |
| Methylbenzoate |  |  | 24.180 | **< 0.001** | 21.90 ± 1.58 | 23.02 ± 2.71 | 10.03 ± 0.65 |
| Phenylacetaldehyde |  |  | 3.272 | 0.051 | 14.66 ± 2.69 | 15.45 ± 2.44 | 8.86 ± 0.92 |
| Phenylethyl alcohol |  |  | 0.281 | 0.757 | 3.87 ± 0.39 | 3.94 ± 0.47 | 3.58 ± 0.50 |
| **Terpenoids** | 7.903 | **< 0.001** |  |  |  |  |  |
| Camphor |  |  | 12.050 | **< 0.001** | 2.65 ± 0.18 | 2.61 ± 0.40 | 1.15 ± 0.16 |
| *E*-α-Farnesene |  |  | 0.428 | 0.655 | 442.1 ± 49.9 | 567.6 ± 103.8 | 472.2 ± 59.0 |
| *Z*-α-Farnesene |  |  | 4.718 | **0.016** | 64.82 ± 9.45 | 55.33 ± 12.84 | 26.81 ± 3.05 |
| **Fatty acid derivatives** | 7.162 | **< 0.001** |  |  |  |  |  |
| *Z*-3-Hexenol |  |  | 1.219 | 0.309 | 133.8 ± 39.9 | 155.6 ± 34.6 | 309.9 ± 140.7 |
| *Z*-3-Hexenyl acetate |  |  | 12.600 | **< 0.001** | 136.5 ± 23.8 | 269.5 ± 62.0 | 1348.0 ± 504.1 |
| Tetradecane |  |  | 2.330 | 0.113 | 47.85 ± 3.21 | 53.91 ± 5.53 | 40.53 ± 3.26 |
| **Nitrogenous compounds** | 12.186 | **< 0.001** |  |  |  |  |  |
| 1-Butene-4-Isothiocyanate |  |  | 2.747 | 0.079 | 644.3 ± 171.6 | 475.4 ± 120.5 | 312.3 ± 102.4 |
| Benzylnitrile |  |  | 22.090 | **< 0.001** | 19.74 ± 2.46 | 12.14 ± 2.01 | 4.31 ± 1.06 |
| Indole |  |  | 1.858 | 0.172 | 7.99 ± 1.12 | 9.76 ± 2.52 | 1.08 ± 2.97 |
| Methylanthranilate |  |  | 172.700 | **< 0.001** | 3.13 ± 0.18 | 0.64 ± 0.07 | 0.33 ± 0.05 |

MANOVA results show a general treatment effect on plant morphology and on floral VOC composition, as well as on all chemical compound classes present in the *B. rapa* scent bouquet. ANOVA on individual traits show that the treatments had an effect on 75% of all measured morphological traits and 53% of all quantified VOC.
