# Supplementary material for: Herbivore-Induced DNA Demethylation Changes Floral Signalling and Attractiveness to Pollinators in Brassica rapa
Source: PLoS One. 2016 Nov 21;11(11):e0166646. doi: 10.1371/journal.pone.0166646 (PMC5117703; doi:10.1371/journal.pone.0166646)
Supplement: S3 Table — (DOCX) [file pone.0166646.s004.docx]

**S3 Table. Phenotypic changes of trait classes in rapid-cycling *B. rapa* plants after 5-azaC treatment.**

|  | **MANOVA 5-azaC treatment** | | **MANOVA genotype** | | **MANOVA treatment × genotype** | |
| --- | --- | --- | --- | --- | --- | --- |
| **Trait class** | ***F*-value** | ***P*-value** | ***F*-value** | ***P*-value** | ***F*-value** | ***P*-value** |
| Morphology | 29.264 | **< 0.001** | 8.169 | **< 0.001** | 2.977 | **< 0.001** |
| Floral VOC | 3.994 | **< 0.001** | 14.548 | **< 0.001** | 1.712 | **0.001** |
|  |  |  |  |  |  |  |
| Aromatics | 4.501 | **< 0.001** | 20.594 | **< 0.001** | 1.604 | **0.034** |
| Terpenoids | 20.425 | **< 0.001** | 8.909 | **< 0.001** | 1.768 | 0.082 |
| Fatty acid derivatives | 5.122 | **0.002** | 3.703 | **< 0.001** | 0.614 | 0.831 |
| Nitrogen-containing compounds | 1.767 | 0.108 | 14.977 | **< 0.001** | 1.343 | 0.127 |

MANOVA results show that both 5-azaC treatment and plant genotype have a significant effect on overall plant morphology and floral VOC emission. Additionally, significant treatment × genotype interactions (t × g) could be observed in both trait groups. Individual MANOVA on different chemical compound classes show that nitrogen-containing compounds do not contribute to the observed treatment effect, and a significant t × g interaction could only be observed for aromatics (*P*-values of significant interactions are in bold).
