# Supplementary material for: Herbivore-Induced DNA Demethylation Changes Floral Signalling and Attractiveness to Pollinators in Brassica rapa
Source: PLoS One. 2016 Nov 21;11(11):e0166646. doi: 10.1371/journal.pone.0166646 (PMC5117703; doi:10.1371/journal.pone.0166646)
Supplement: S4 Table — (DOCX) [file pone.0166646.s005.docx]

**S4 Table. Phenotypic differences between rapid-cycling *Brassica rapa* plants under control and 5-azacytidine treatment.**

|  | **Mean ± 1 SE** | | **Treatment** | **ANOVA treatment** | | **ANOVA genotype** | | **ANOVA tre. × gen.** | |
| --- | --- | --- | --- | --- | --- | --- | --- | --- | --- |
| **Plant trait** | **Control** | **5-azaC Treatment** | **effect** | ***F*-value** | ***P*-value** | ***F*-value** | ***P*-value** | ***F*-value** | ***P*-value** |
| **Morphological Traits** |  |  |  |  |  |  |  |  |  |
| Plant Height [cm] | 23.16 ± 0.38 | 17.56 ± 0.44 | ↓ | 120.986 | **< 0.001** | 6.608 | **< 0.001** | 9.148 | **< 0.001** |
| Time to flowering [d] | 18.39 ± 0.10 | 19.32 ± 0.11 | ↓ | 46.875 | **< 0.001** | 11.118 | **< 0.001** | 2.864 | **0.025** |
| Leaf Number | 10.71 ± 0.30 | 7.98 ± 0.24 | ↓ | 69.038 | **< 0.001** | 6.407 | **< 0.001** | 8.655 | **< 0.001** |
| Bud Number | 41.52 ± 2.06 | 21.21 ± 1.34 | ↓ | 114.612 | **< 0.001** | 2.445 | **0.048** | 13.474 | **< 0.001** |
| Flower Number | 8.22 ± 0.25 | 7.07 ± 0.20 | ↓ | 14.520 | **< 0.001** | 8.123 | **< 0.001** | 4.237 | **0.003** |
| Flower Petal Area [mm^2^] | 84.72 ± 1.66 | 67.45 ± 1.36 | ↓ | 105.353 | **< 0.001** | 25.438 | **< 0.001** | 7.497 | **< 0.001** |
| Plant Petal Area [mm^2^] | 704.20 ± 27.31 | 485.50 ± 19.51 | ↓ | 59.871 | **< 0.001** | 16.286 | **< 0.001** | 6.803 | **< 0.001** |
| Nectar Amount [μl] | 5.52 ± 0.26 | 4.28 ± 0.24 | ↓ | 15.263 | **< 0.001** | 2.456 | **< 0.001** | 4.513 | **0.002** |
| Pollen [grains / flower] | 67.13 ± 3.79 | 61.83 ± 3.98 | - | 2.139 | 0.145 | 2.281 | 0.062 | 1.017 | 0.400 |
| **VOC** [pg / flower l^-1^] |  |  |  |  |  |  |  |  |  |
| **Aromatics** |  |  |  |  |  |  |  |  |  |
| *p*-Anisaldehyde | 24.28 ± 3.81 | 16.66 ± 2.22 | ↓ | 7.104 | **0.008** | 56.757 | **< 0.001** | 0.380 | 0.823 |
| Benzaldehyde | 402.90 ± 23.59 | 397.35 ± 26.88 | - | 0.355 | 0.552 | 4.589 | **0.001** | 0.913 | 0.458 |
| Methylbenzoate | 124.58 ± 10.44 | 88.92 ± 6.39 | ↓ | 14.407 | **< 0.001** | 26.106 | **< 0.001** | 0.922 | 0.452 |
| Methylsalicylate | 58.64 ± 8.52 | 53.81 ± 5.34 | - | 0.141 | 0.708 | 44.459 | **< 0.001** | 1.670 | 0.159 |
| Phenylacetaldehyde | 304.12 ± 41.59 | 266.67 ± 38.67 | - | 2.515 | 0.114 | 51.685 | **< 0.001** | 1.724 | 0.146 |
| Phenylethyl alcohol | 11.88 ± 1.47 | 9.94 ± 1.66 | ↓ | 9.226 | **0.003** | 27.316 | **< 0.001** | 3.158 | **0.015** |
| **Terpenoids** |  |  |  |  |  |  |  |  |  |
| *E*-α-Farnesene | 1406.36 ± 79.12 | 1010.94 ± 65.89 | ↓ | 20.141 | **< 0.001** | 18.364 | **< 0.001** | n/a | n/a |
| *Z*-α-Farnesene | 58.44 ± 3.09 | 38.29 ± 2.18 | ↓ | 33.420 | **< 0.001** | 11.754 | **< 0.001** | n/a | n/a |
| **Fatty acid derivates** |  |  |  |  |  |  |  |  |  |
| *Z*-3-Hexenyl acetate | 95.87 ± 9.89 | 79.00 ± 5.96 | ↓ | 4.045 | **0.046** | 6.650 | **< 0.001** | n/a | n/a |
| Tridecane | 10.85 ± 0.67 | 12.06 ± 1.01 | - | 0.003 | 0.958 | 2.533 | **0.042** | n/a | n/a |
| Tetradecane | 82.48 ± 3.45 | 78.24 ± 3.95 | - | 2.198 | 0.140 | 4.001 | **0.004** | n/a | n/a |
| **Nitrogenous compounds** |  |  |  |  |  |  |  |  |  |
| 1-Butene-4-Isothiocyanate | 84.94 ± 15.33 | 66.25 ± 8.82 | n/a | n/a | n/a | 8.353 | **< 0.001** | n/a | n/a |
| 6-Met.-2- Pyridinecarbaldehyde | 799.55 ± 80.48 | 539.91 ± 52.96 | n/a | n/a | n/a | 8.118 | **< 0.001** | n/a | n/a |
| Benzylnitrile | 152.37 ± 15.88 | 119.82 ± 11.66 | n/a | n/a | n/a | 31.975 | **< 0.001** | n/a | n/a |
| Indole | 166.92 ± 14.07 | 129.02 ± 12.26 | n/a | n/a | n/a | 11.124 | **< 0.001** | n/a | n/a |
| Formanilide | 9.69 ± 0.73 | 9.93 ± 0.95 | n/a | n/a | n/a | 2.476 | **0.046** | n/a | n/a |
| Methylanthranilate | 235.31 ± 26.96 | 155.40 ± 16.04 | n/a | n/a | n/a | 4.550 | **0.002** | n/a | n/a |

ANOVA on individual traits show that the 5-azacytidine treatment had an effect on 89% of all measured morphological traits and 35% of all quantified VOC. All significant traits were negatively affected, and 9 of these traits showed significant treatment × genotype interactions. Except for pollen quantity, plant genotype had a significant impact on all measured phenotypic traits irrespective of the demethylation treatment. Nitrogen compounds had no significant effect in the MANOVA analysis and were therefore excluded from the analysis*.*  Arrow up: trait increase, arrow down: trait decrease, minus: no trait change, *P* value of significant changes in bold (α = 0.05).
